# Evaluation of the Possible Influence of Povidone Iodine (PVI) Solution and Polyhexanide (PHMB) on Wound Healing in Corneal Epithelial Regeneration

**DOI:** 10.3390/jcm13020588

**Published:** 2024-01-19

**Authors:** Sabine Foja, Joana Heinzelmann, Anja Viestenz, Christiane Rueger, Sabine Hecht, Arne Viestenz

**Affiliations:** Department of Ophthalmology, University Hospital, Martin-Luther-University Halle-Wittenberg, 06120 Halle (Saale), Germany; joana.heinzelmann@uk-halle.de (J.H.); sabine.hecht@uk-halle.de (S.H.);

**Keywords:** preoperative antisepsis, povidone iodine (PVI), polyhexanide (PHMB), Serasept^®^ 2, cornea, wound healing

## Abstract

This study investigates the possible toxic effects of the preoperative antiseptic substances povidone iodine (PVI) and polyhexanide (PHMB; Serasept^®^ 2) on wound healing in ophthalmology. To assess this impact, human telomerase-immortalized corneal epithelial (hTCEpi) cells and human telomerase-immortalized conjunctival epithelial (hCjE) cells were exposed to 1% and 5% PVI or 0.04% PHMB for different periods to evaluate the cytotoxicity of these two antiseptics. Furthermore, the toxicity of these antiseptics was investigated in a human tissue-specific corneal epithelial construct and porcine eye culture model. The results reveal the high cytotoxicity of PVI and PHMB in the hTCEpi and hCjE in monolayer cell culture models, independent of the incubation time and concentration of these substances. However, after hTCEpi cell differentiation into a tissue-specific corneal epithelial construct, contact with these antiseptics for the relevant preoperative time did not alter cPARP1 or Ki67 expression. Furthermore, the wound-healing process in the porcine cornea was not significantly influenced after incubation with these antiseptics. In summary, corneal and conjunctival epithelial cell lines are very sensitive to PVI and PHMB, whereas no significant alterations were found in intact tissue-specific corneal epithelial constructs or porcine corneas. Therefore, we could not identify PVI and PHMB as reasons for postoperative eye irritation.

## 1. Introduction

Postoperative endophthalmitis, an infection of the interior cavity of the eye, is a serious complication of ocular surgery that can lead to a reduction in visual acuity and even loss of the eye itself. In an effort to minimize the risk of endophthalmitis after ophthalmic surgery, prophylactic steps are necessary to reduce the number of microorganisms. Therefore, povidone iodine (also known as polyvinylpyrrolidone iodine, PVI) and polyhexanide (also known as polyhexamethylene biguanide, PHMB) as alternative agents are used in the pre- and perioperative periods to minimize the quantity of microorganisms on the ocular surface [1,2,3,4,5].

PVI has been widely used for endophthalmitis prophylaxis. PVI is composed of diatomic iodine and polyvinylpyrrolidone (povidone). Povidone is a water-soluble polymer that serves as a carrier for iodine; 10% PVI solution contains 1% free available iodine. Iodine exhibits its microbicidal effect by oxidizing water to release ions that directly act on bacterial or viral membrane proteins [6]. Since iodine also acts directly on membrane proteins of eukaryotic cells, the use of effective and nontoxic concentrations for an appropriate contact time is important. Nevertheless, to date, different concentrations ranging from 0.025% to 10% PVI and incubation times varying from seconds to minutes have been used worldwide [6,7].

In the rare case of an allergic reaction to PVI, PHMB may be used as an alternative agent. PHMB interacts with acidic phospholipids in microbial membranes, resulting in their disruption, while the neutral phospholipids in human cell membranes are only marginally affected. The antimicrobial agent PHMB is available commercially in different formulations. The most common formulation is Lavasept^®^, a combination of PHMB with macrogol, and Serasept^®^ 2, which comprises PHMB in Ringer solution DAB 7 [8].

In a study that included more than 13,000 patients treated with intravitreal injections, PHMB was effective against microbes, with a resulting incidence of endophthalmitis comparable to that of PVI in previous studies [2]. The authors summarized that PHMB is less irritating to the ocular surface, resulting in less discomfort and pain than PVI. However, preoperatively, PHMB requires a longer exposure time than PVI. In this study, 0.04% PHMB was applied three times in intervals of ten minutes. According to Hansmann et al. [3], for preoperative antiseptic prophylaxis, PHMB should be applied for 10 min to develop its antimicrobial activity.

According to the literature and our experience, after successful surgery, corneal damage and symptoms of irritation, including painful dry eye, occur. Therefore, the possible toxicity of these antiseptic substances to the ocular surface, including limbal stem cells, in a time- and concentration-dependent manner has been discussed. However, to date, evidence and consensus remain lacking [9,10,11].

Therefore, basic and clinical research, including in vitro cytotoxicity assays, are important for evaluating the potential toxicity of antiseptic substances used in the preoperative surgical setting and the possible risk of associated corneal damage, including a possible association with symptoms of irritation in patient eyes.

In the present study, we evaluated the corneal toxicity of PVI (1% and 5%) and PHMB (0.04%) using two epithelial cell lines of corneal and conjunctival origin, i.e., human telomerase-immortalized corneal epithelial (hTCEpi) cells and human telomerase-immortalized conjunctival epithelial (hCjE) cells. Using both cell lines, we investigated possible toxic effects on cells that are in contact with antiseptic substances during surgery. Therefore, we investigated the proliferation and colony-forming efficiency of the limbal epithelial stem cell line as well as morphological alterations in cultured cells. Furthermore, we used 3D differentiated hTCEpi cells to validate these results regarding apoptosis and proliferation capacity. Additionally, the effects of wound healing were analyzed in an ex vivo cultured porcine cornea model.

## 2. Materials and Methods

### 2.1. Cell Culture

The hTCEpi cell line is derived from corneal tissue in the limbal region, expresses stem cell markers, and has the ability to differentiate into a 3D tissue-specific corneal epithelial cell construct. The hCjE cell line is sourced from the conjunctival region. hTCEpi cells were maintained in keratinocyte growth medium (KGM)-2 supplemented with KGM-2 SingleQuot Kit supplements (Lonza, Basel, Switzerland). hCjE cells were cultured in keratinocyte serum-free medium (SFM) supplemented with epidermal growth factor (EGF) and bovine pituitary extract (BPE) (Gibco, Germany). The cells were cultured in a 5% CO_2_ humidified atmosphere at 37 °C and passaged every 3 to 5 days according to the standard operation procedure described in the manufacturer’s instructions. Authentication of the cell lines was performed.

### 2.2. Differentiation into Tissue-Specific Corneal Epithelial Constructs and Treatment

For differentiation, hTCEpi cells were submerged in high-calcium KGM-2. Therefore, 1.7 × 10^4^ hTCEpi cells were cultured in the upper well of a transwell system using ThinCert culture inserts (24-well, 0.4 µm pore size, Greiner Bio-One GmbH, Frickenhausen, Germany) with KGM-2 containing 1.15 mM Ca^2+^. The medium was changed daily for 7 days. On day 8, cells in the upper well were airlifted by removing the medium from the inserts. Cells were cultured for an additional 14 days by renewing the medium in the lower part every day. Cells were maintained in 5% CO_2_ in a humidified atmosphere at 37 °C. On day 21, hTCEpi cells were treated with the longest incubation time and the highest concentration of each antiseptic substance, i.e., 5% PVI (povidone iodine, manufactured in a clinical pharmacy, Halle-Saale, Germany) for 2 min and 0.04% PHMB (polyhexanide, polyhexamethylene biguanide, Serasept^®^ 2, SERAG-Wiessner GmbH, Naila, Germany) for 30 min. Afterward, tissues were rinsed 3 times and were cultured for an additional 48 h. Inserts with cells were fixed in 4% paraformaldehyde (PFA) for 24 h for hematoxylin and eosin (H&E) and periodic acid-Schiff (PAS) staining as well as for Ki67 and cPARP1 expression analyses.

### 2.3. Organ Culture of Porcine Eyes

Fresh porcine eyes were obtained from a slaughterhouse (Tönnies, Weißenfels, Germany). The procedure for eye preparation was modified as described by Castro et al. [12]. In brief, eyes were collected within 3 h of extraction and transported in phosphate-buffered saline (PBS) containing 10% fetal calf serum (FCS) and 1% antibiotic antimycotic solution (ABAM, Thermo Fisher Scientific, Karlsruhe, Germany) on ice to the laboratory and processed immediately. After removing extraocular tissue, the bulbs were washed in sterile PBS, decontaminated with 2% PVI three times, and washed again twice in PBS. Two different approaches were applied. First, intact globes were treated with antiseptic substances at different concentrations and times to evaluate the cytostatic impact on intact corneas. In the second group, the effect on corneal wound-healing capacity was investigated. Therefore, a corneal wound was created before treatment with antiseptic substances. To create the wound, the central corneal epithelium was completely removed using a hockey knife without disturbing the limbus. Bulbs in both approaches were treated either with 1% PVI for 1 min, 5% PVI for 2 min, or 0.04% PHMB for 3 min and 30 min. In both groups, untreated control bulbs were used.

Corneas were separated from the globe, with a small rim of scleral tissue remaining. To support the native corneal structure under culture conditions, the cornea was filled with warmed sterile 1% agar in Dulbecco’s Modified Eagle Medium F12 (DMEM/F12, Fisher Scientific, Germany) and 1 mg/mL bovine collagen (CellSystems, Troisdorf, Germany).

Corneas were cultured in 6-well plates containing 5 mL of DMEM/F12 supplemented with 1% ITS liquid media supplement, 1% RPMI 1640 vitamin solution, 1% ABAM, 0.5% gentamycin 1% sodium pyruvate (Merck, Darmstadt, Germany), 1 µg/mL glutathione, 1% MEM non-essential amino acids solution (ThermoFisher Scientific, Karlsruhe, Germany), 20 mM L-glutamine (Fisher Scientific, Schwerte, Germany), and 0.1% vitamin C (Sigma, Taufkirchen, Germany) under airlifting conditions. The medium was refreshed daily. Simultaneously, the corneal surfaces were moisturized with media every day.

To evaluate the epithelial wound-healing capacity depending on the treatment option, fluorescein corneal staining was performed using Thilorbin (OmniVision, Puchheim, Germany) on days 0, 2, and 5. For the detection of wound areas, corneas were coated with 1–2 fluorescein-containing eye drops and washed three times with 1 mL PBS. Epithelial defect areas were intensely stained, enabling a clear differentiation from the darker intact regions. The wound healing area was detected by generating fluorescence images of the corneal surface using an Axio Zoom V16 microscope (Zeiss, Oberkochen, Germany), and the area was measured using the microscopy software Zen blue (Zen 2.5, Version 2.5.75.0, Zeiss, Oberkochen, Germany). To calculate the relative wound-healing capacity, the differences between the green fluorescence region on day 0 and day 5 were calculated (wound healing area = measured fluorescence wound area on day 0—measured fluorescence wound area on day 5). The wound healing area of the medium control group on day 5 was established as the baseline at 100%, and all other groups were compared relative to it. After 5 days of cultivation, corneas were fixed in 4% PFA over 48 h for H&E and PAS staining. Experiments were repeated three times, with two corneas twice and then with three corneas in each group (n = 7).

### 2.4. Colony-Forming Assay

For the colony-forming assay, 250 hTCEpi cells were plated on 100 × 20 mm cell culture dishes (Saarstedt AG, Nümbrecht, Germany) and cultured in KGM-2 at 37 °C in a humidified atmosphere. On day 2, the cells were treated with 1% and 5% PVI or 0.04% PHMB for different periods of time, washed three times with BSS each for 1 min, and cultured for an additional 12 days. An untreated control group and a BSS-rinsing control group were also used. Cell colonies were fixed with 4% PFA for 30 min (Thermo Fisher Scientific, Germany) and were irrigated with PBS. Before colony counting, cells were stained with Congo red for 15 min (Carl Roth, Karlsruhe, Germany) at room temperature. To obtain the colony-forming efficiency (CFE), the following equation was used: CFE = number of colonies per plate/number of seeded cells × 100. This experiment was performed in three different biological replicates.

### 2.5. Cell Viability

HTCEpi and hCjE cells were seeded at a density of 5 × 10^3^ cells in 24-well plates and incubated for 48 h. Afterward, cultured cells were incubated with 1% and 5% PVI for 1 and 2 min or with 0.04% PHMB for 3 min and 30 min, washed with BSS three times for 1 min each, and cultured for an additional 48 h.

Two control groups were included. In the BSS control group, cells were flushed 3 times as in the treatment group. In the medium control group, cells were cultured without treatment or washing steps.

The CellTiter-Glo (Promega, Walldorf, Germany) cell viability assay was performed according to the manufacturer’s instructions. In brief, CellTiter-Glo reagent was equilibrated to room temperature. After adding CellTiter-Glo to the cells, the plates were vigorously mixed for 5 min. After an additional 25 min of incubation, the luminescence was detected using an Infinite 200 reader (Tecan, Nänikon, Switzerland).

Experiments were performed in triplicate three times. The results from the viability assays are expressed as the percentage of induced cytotoxicity compared to the nontreated control.

### 2.6. Immunohistochemistry and Microscopy

The tissue-specific corneal epithelial constructs and porcine corneas were fixed in 4% PFA for 48 h, followed by storage in 70% ethanol. The tissues were embedded in paraffin using a histokinette (Leica, Wetzlar, Germany). Tissues were cut into 4 µm sections using a rotary microtome (Leica, Germany).

After antigen retrieval, the samples were blocked with 0.1% TBST containing 20% bovine serum albumin (BSA) and 5% FCS for 30 min and incubated with primary antibodies against Ki67 (RM-9106-5; clone SP6, Thermo Scientific, Germany, dilution 1:100) and anti-cleaved PARP1 (cPARP1) (ab32064, Abcam, Berlin, Germany, dilution 1:1000) overnight at 4 °C. Antibody binding was detected by incubation with Alexa Fluor 488-conjugated secondary antibodies (4412S, Cell Signaling Technology, Frankfurt am Main, Germany) for 1 h at room temperature. Nuclear counterstaining and mounting were performed using Prolong Gold antifade reagent with DAPI (Invitrogen by Thermo Fischer Scientific, Karlsruhe, Germany). As negative controls, the primary antibodies were replaced by isotypic primary antibodies. Histologic H&E and PAS staining were performed routinely at the Institute of Pathology and documented with light microscopy (Zeiss, Oberkochen, Germany).

For determination of population growth kinetics, slides were scanned (Axion Scan Z1 Scanner, Oberkochen, Zeiss). The number of Ki67-positive cells (proliferating cells), cPARP1-positive cells (apoptotic cells), and DAPI-stained cells in tissue-specific corneal epithelial differentiated hTCEpi cell constructs were counted by two independent investigators under blinded conditions. The experiments were performed in duplicate in two independent approaches.

### 2.7. Statistical Analyses

Statistical analyses for calculation of altered Ki67 and cPARP1 expression in tissue-specific corneal epithelial constructs as well as for the calculation of wound healing in the pig eye samples were performed using the *T* Test. The level of significance was set at 5%. 

The results of the *t*-test were subjected to verification through the application of an analysis of variance (ANOVA) test. Analyses were performed using the statistical program SPSS for Windows (SPSS, Inc. (Chicago, IL, USA), version 28).

## 3. Results

### 3.1. Cytotoxic Effects of Antiseptic Agents on Corneal and Conjunctival Epithelial Cells In Vitro

**Figure 1:**
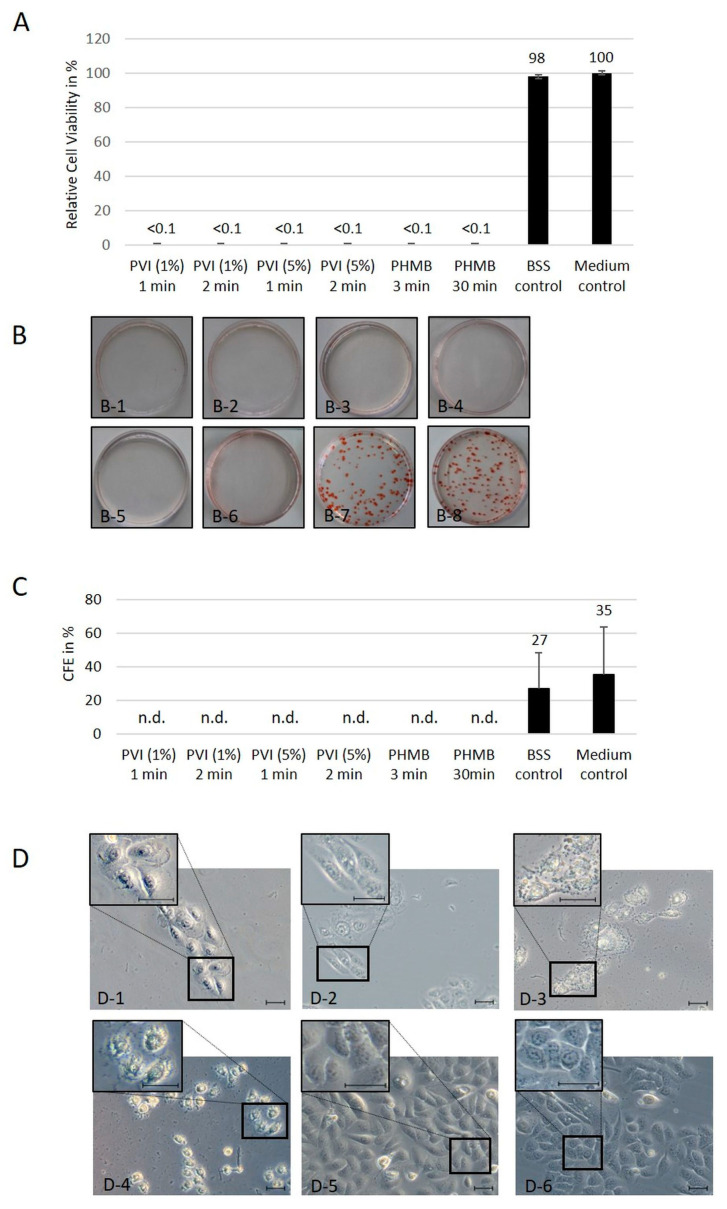
Cytotoxic effects of antiseptic agents on a human corneal epithelial cell line (hTCEpi): (**A**) Relative cell viability of human hTCEpi cells following exposure to 1% and 5% PVI and 0.04% PHMB for different incubation times and culture for additional 48 h after treatment. The mean values and standard deviations represent data from three separate experiments, each performed in triplicate. (**B**) Images of the colony-forming assay results 10 days after treatment based on 250 seeded cells per dish. On day 2, the cells were treated with antiseptic agents for different times. Dishes were fixed on day 12 and stained with Congo red. Shown are the results after treatment with 1% PVI for 1 min (**B-1**), 1% PVI for 2 min (**B-2**), 5% PVI for 1 min (**B-3**), 5% PVI for 2 min (**B-4**), 0.04% PHMB for 3 min (**B-5**) and 30 min (**B-6**) as well as the controls after 3 washes with BSS (**B-7**) and culture in medium (**B-8**). (**C**) Calculation of colony-forming efficiency (CFE). The mean values and standard deviations represent data from three separate experiments (n.d. = nondetectable). (**D**) Typical hTCEpi cell morphology observed on day 2 after treatment with 1% PVI for 1 min (**D-1**), 5% PVI for 2 min (**D-2**), 0.04% PHMB for 3 min (**D-3**), and 0.04% PHMB for 30 min (**D-4**) as antiseptic substances compared to BSS rinsing (**D-5**) and culture in medium (**D-6**) as controls. Scale bars represent 20 µm.

**Figure 2:**
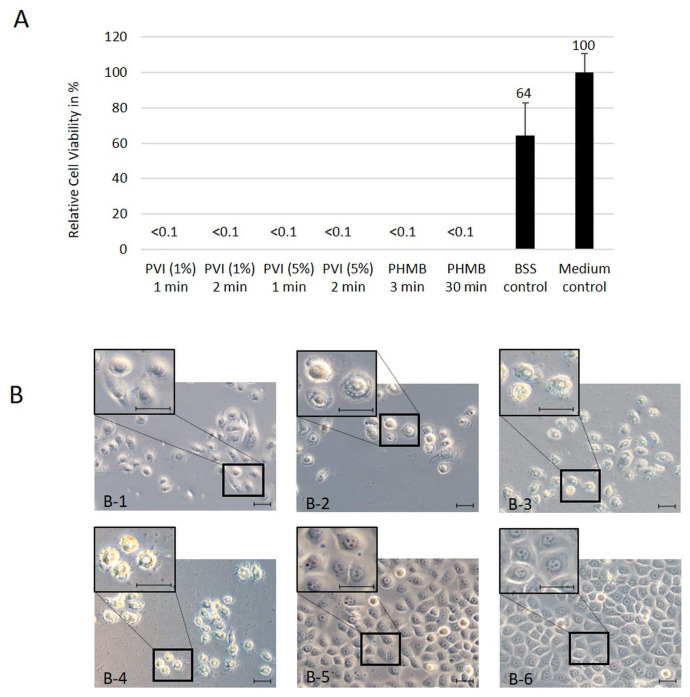
Cytotoxic effects of antiseptic agents on a human conjunctival epithelial cell line (hCjE): (**A**) Cell viability assay after treatment of human conjunctival epithelial (hCjE) cells with the antiseptic substances PVI and 0.04% PHMB. The mean values and standard deviations represent data from three separate experiments, each performed in triplicate. (**B**) Typical hCjE cell morphology observed on day 2 after treatment with antiseptic substances 1% PVI for 1 min (**B-1**), 5% PVI for 2 min (**B-2**), 0.04% PHMB for 3 min (**B-3**), and 0.04% PHMB for 30 min (**B-4**) compared to BSS rinsing (**B-5**) and culture in medium (**B-6**) as controls. Scale bars indicate 20 µm.

HTCEpi and hCjE cells were very sensitive to 1% and 5% PVI as well as 0.04% PHMB. In the cell culture experiments, both antiseptic substances were cytotoxic to more than 99% of corneal epithelial cells (Figure 1A). This effect was observed independently of incubation time and substance concentration. Therefore, as shown in Figure 1A, hTCEpi cell viability was decreased to under 0.1% after exposure to 1% and 5% PVI for 1 min and 2 min or 0.04% PHMB for 3 min and 30 min compared to the untreated control cells. Exposure of hCjE cells to these antiseptic substances confirmed the results, with a viability rate lower than 0.1%, independent of the utilization of PVI or PHMB as well as the incubation time and substance concentration (Figure 2A).

**Figure 3:**
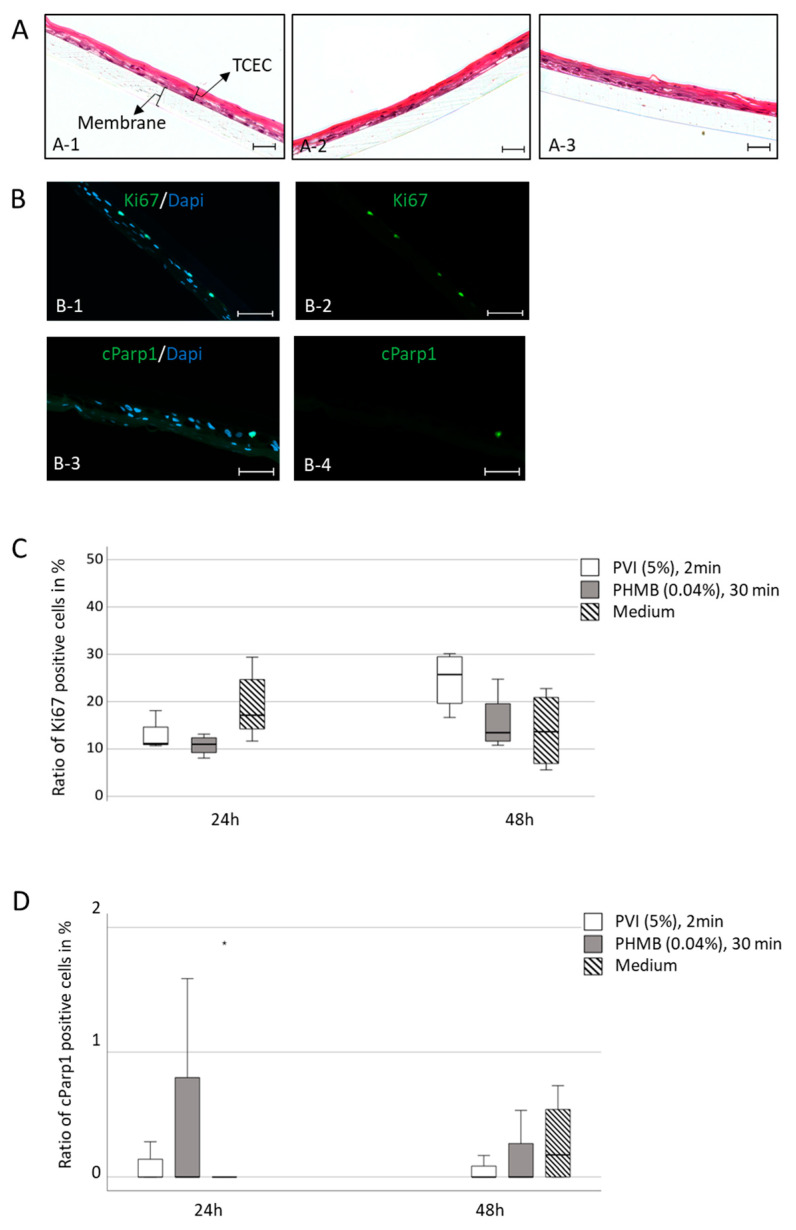
Immunohistochemical analyses of differentiated hTCEpi tissue-specific constructs after exposure to PVI and PHMB: (**A**) H&E staining after differentiation of hTCEpi cells over 21 days; treatment with 5% PVI for 2 min (**A-1**) and 0.04% PHMB for 30 min (**A-2**) and further culture for 48 h. The untreated control is shown in (**A-3**). Scale bars represent 20 µm. (TCEC = tissue-specific corneal epithelial construct). (**B**) Immunohistochemical images of hTCEpi differentiated tissue- specific constructs in merged pictures: (**B-1**,**B-3**) blue-nuclei staining and green-fluorescence of Ki67 or cParp1; (**B-2**,**B-4**) the green fluorescence for positive Ki67 or cParp1 staining cells. Scale bars indicate 50 µm. (**C**) The percentage of Ki67-positive cells was calculated after treatment of a 3D tissue-specific corneal epithelial construct with 5% PVI for 2 min and 0.04% PHMB for 30 min, with flushing and further culture for 24 h and 48 h. Data represent two separate experiments, each performed in duplicate. (**D**) The percentage of cParp1-positive cells was calculated after treatment of a 3D tissue-specific corneal epithelial construct with antiseptic substances, flushing and further culture for 24 h and 48 h. Data represent two separate experiments, each performed in duplicate. (* represents extreme outlier- values more than 3 times the interquartile range).

**Figure 4:**
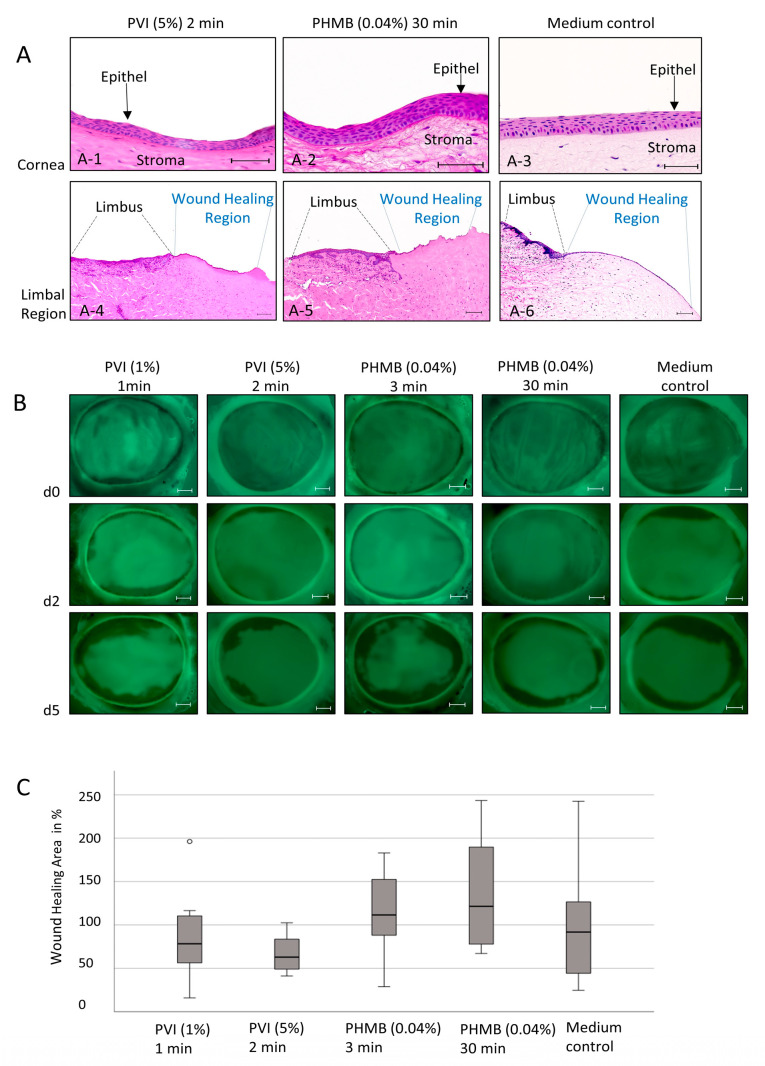
Wound-healing analyses after exposure of ex vivo cultured porcine corneas to PVI and PHMB: (**A**) H&E staining of pig corneal epithelia and the limbal region on day 5 after treatment with 5% PVI for 2 min, 0.04% PHMB for 30 min, or medium as a control. Scale bars indicate 50 µm (**A-1**–**A-3**) and 100 µm (**A-4**–**A-6**). (**B**) Fluorescein staining of porcine eyes on day 0, day 2, and day 5 after induction of complete corneal erosion and treatment with antiseptic substances. Scale bars indicate 2000 µm. (**C**) Wound healing area (%) of porcine corneas after treatment with antiseptic substances in relation to the untreated control group on day 5 of culture. Data represent results from three independent experiments, with n = 7–9 eyes per treatment group. No significant results were found. (° represents mild outlier- values more than 1.5 times the interquartile range).

Light microscopy analysis demonstrated further morphological alterations, with destruction of the structure of the hTCEpi (Figure 1D) and hCjE cells (Figure 2B). Forty-eight hours after exposure to PHMB for 3 min and 30 min, strong blebbing of the membrane structure as well as a large amount of cellular debris in the intercellular space were observed in both cell lines (Figure 1D(D-3,D-4) and Figure 2B(B-3,B-4)). After application of 1% and 5% PVI, no visible disruption of the cellular membrane was observed, but cells appeared strongly flattened, with dominant nuclei and strongly reduced cytoplasmic content in both cell lines (Figure 1D(D-1,D-2) and Figure 2B(B-1,B-2)).

Limbal stem cells have the ability to form tight, compact colonies. Therefore, the number of colony-forming cells has often been used as a surrogate for evaluating the number of intact stem cells contained in a culture. We therefore analyzed the CFE after treating hTCEpi cells with 1% PVI for 1 min and 2 min, with 5% PVI for 1 min and 2 min, and with 0.04% PHMB for 3 min and 30 min. The median CFE of the untreated control group was 35%. In the BSS-treated control group, the median CFE was 27% due to flushing of the cells with BSS.

After treatment with the antiseptic substances PVI and PHMB, no colonies were detected, independent of substance concentration and incubation time. In summary, no cells with colony-forming capacity survived the treatment, even after the shortest exposure time of 1 min with 1% and 5% PVI and 3 min with 0.04% PHMB (Figure 1B).

### 3.2. Viability of Human Tissue-Specific Corneal Epithelial Construct

With an increased calcium concentration and under airlifting conditions, hTCEpi cells differentiate into a tissue-specific corneal epithelial construct that reflects typical characteristics of the human corneal epithelium, including basal cells, wing cells, and the surface layer [13]. Therefore, differentiated hTCEpi cells were used as a culture model for the corneal epithelium and were incubated with PVI and PHMB in accordance with the surgical procedure. Even 48 h after exposure to the highest concentration of antiseptic substances over the longest incubation time, an intact surface, including the wing and basal cell layers, was observed (Figure 3A). Therefore, we could not find morphological alterations due to the application of antiseptic substances in comparison with the untreated control group.

Furthermore, no significant changes in the concentration of the proliferation marker Ki67 were found in tissue-specific corneal epithelial constructs after treatment with antiseptic substances and culture for 24 h or 48 h (Figure 3B,C). Moreover, the incubation of tissue-specific corneal epithelial constructs with the antiseptic substances PVI and PHMB had no significant influence on the expression of the apoptotic marker cPARP1 (Figure 3D).

### 3.3. Wound Healing and Morphology of the Epithelium and Limbus in Organ Cultures

Intact porcine corneas were used as an ex vivo model to analyze the cytotoxic effect of the antiseptic agents (1% and 5% PVI as well as 0.04% PHMB) on corneal epithelial tissue under conditions similar to those in vivo. In all treatment groups, intact basal cell layers, wing cells, and surface cells could be identified 5 days after treatment. In summary, after treatment with 1% and 5% PVI and 0.04% PHMB, no significant loss or destruction of the epithelial layer could be observed, even with long incubation times, as shown in Figure 4A-1 up to Figure 4A-3.

Furthermore, the effect of antiseptic agents on wound healing was investigated after complete removal of the corneal epithelium of the pig eyes up to the limbal region. As shown in the injured corneas, crypt-like structures of the limbal region were preserved after treatment with 5% PVI for 2 min and 0.04% PHMB for 30 min and a culture time of 5 days (Figure 4A(A-4–A-6)). The wound-healing capacity was determined using H&E staining (Figure 4A) as well as fluorescein staining (Figure 4B).

We observed the epithelial wound-healing ability of the cornea 5 days after treatment with 1% PVI for 1 min, 5% PVI for 2 min, and 0.04% PHMB for 3 min and 30 min (Figure 4B). By calculating the area of wound healing 5 days after treatment, we did not find significant differences depending on the application of the different antiseptic substances (Figure 4C).

## 4. Discussion

A key objective of this study was to verify the potential cytotoxic effects of the antiseptic substances PVI and PHMB on the corneal epithelium, depending on concentration and exposure time as possible causes of observed corneal damage and symptoms of irritation after successful surgery. Both substances are normally used in surgery. Unfortunately, to date, no consistent concentrations and incubation times have been defined. Therefore, the effective concentrations of PVI regarding efficiency in reducing the bacterial contamination rate have been reported to range from 0.025 to 10% [7,14]. According to the guidelines of the European Society of Cataract and Refractive Surgery, a 5% to 10% PVI solution should be applied to the cornea, conjunctival sac, and periocular skin for 3 min prior to surgery [15]. However, lower concentrations of PVI are common. To prevent bacterial contamination and endophthalmitis, Shimada et al. [16] recommended the use of a 1.25% PVI solution for preoperative preparation after analysis of the surgical outcomes of more than 4000 eyes subjected to vitrectomy. Silas et al. [4] reported the repeated use of 1% PVI for 90 s as effective for preoperative antisepsis in an experimental study. Lindquist et al. [1] evaluated the influence of 2 min of exposure to 1% and 5% PVI for conjunctival lavage in corneal donors. According to his investigation, the use of a 1% PVI solution for corneal donor preparation was recommended. This issue seems to be particularly significant in corneal transplantation, in which contamination of the donor cornea can increase the risk of endophthalmitis [1].

In the case that PVI is contraindicated, alternative substances such as PHMB may be used. In a study from the University Hospital of Cologne, 0.04% PHMB was used in more than 13,000 intravitreal injections between January 2007 and September 2013 for preoperative antisepsis; it was applied three times for an interval of ten minutes [2].

It is important to note that antiseptic prophylaxis and antimicrobial activity are the main purposes for identifying an effective antiseptic concentration and exposure time before surgery. Nevertheless, it is possible that antiseptic substances used during ophthalmic procedures could be pathogenic factors that damage the cornea and cause wound-healing disorders. Therefore, ocular surface damage due to exposure to 5% PVI in a time-dependent manner (incubation up to 10 min) was investigated in rabbits [9]. Additionally, epithelial damage was observed 30 min after the instillation of 5% PVI into the conjunctival sac in a rabbit model [5]. In cultured human corneal fibroblasts, damage was observed after exposure to PVI and PHMB in correlation with increased concentrations and exposure times [10,17,18].

Therefore, we used a 2D cell culture model to investigate the direct cytotoxic effects of antiseptic substances on epithelial cells. After treatment of the cultured human corneal epithelial cells and conjunctival epithelial cells with PVI and PHMB, we observed several patterns of pathological morphological alterations. The application of PVI resulted in flat cell structures with very low cytoplasm content, comparable with that of fixed cells. Treatment with PHMB caused strong membrane blebbing and the accumulation of a large amount of cell debris in the intercellular space. These observations could be explained by different principles of action. The free iodine from PVI penetrates into cells and oxidizes key proteins, nucleotides, and fatty acids, eventually leading to cell death [19,20]. However, PHMB perforates membranes, disrupting the cell structure and precipitating intracellular constituents. Once inside the cell, the cationic polymer can selectively condense microbiological chromosomes, which may block the DNA replication process of bacteria. To date, the mechanism of action remains not fully understood [20,21].

A strong cytotoxic effect of PVI and PHMB independent of the analyzed concentrations and incubation times was found in 2D cell culture. These results are in agreement with those of the colony formation assay using hTCEpi cells, which express limbal stem cell markers and have the ability to form tight, compact colonies. Therefore, the number of colony-forming cells has been used as a surrogate for evaluating the number of intact stem cells. After treatment with the antiseptic substances PVI and PHMB, no colonies could be detected, independent of antiseptic substance, treatment time and substance concentration.

Our observations in the 2D cell culture model correlate with those of previous publications [10,18]. Pels et al. [18] found significant damage to corneal fibroblasts after exposure to 0.25% PVI for 2 min. Nearly total damage was observed at a concentration of 1% PVI directly and 48 h after immersion. Shibata et al. [10] concluded that cytotoxicity from PVI in cell culture could be explained by the available iodine concentration and partly by its pH, surfactant, and osmolality. The toxicity of PVI and PHMB has also been demonstrated in vitro by Yanai et al. [17], who tested PVI from 0.0125% to 0.25% and PHMB from 0.001% to 1%, with an incubation time of up to 30 min in human cultured corneal epithelial cells, and showed toxicity to be enhanced with both increasing concentration and exposure time.

However, we did not observe any cytotoxic alterations in the expression of apoptotic (cParp1) or proliferative (Ki67) markers in a tissue-specific in vitro model or ex vivo porcine cornea model, including changes in morphological structures or wound-healing capacity after exposure to the antiseptic substances PVI and PHMB. Therefore, the anatomical and physiological barriers of the cornea seem to protect the multilayered eye structure, leading to different results in the 3D cornea tissue-specific model and porcine cornea model than the 2D cell culture model. In these multilayered structures, no toxic effects of antiseptic substances were observed.

The observed nontoxic effects of the antiseptic substances on the tissue-specific constructs as well as porcine corneas agree with observations of rabbit eyes as reported by Jiang et al. [5]. The authors postulated that PVI solution has difficulty penetrating through the corneal surface in rabbit eyes. They observed that concentrations of PVI lower than 2.5% applied in the conjunctival sac are safe and not toxic to epithelial cells; only the instillation of 2.5% and 5% PVI into rabbit eyes with 30 min of exposure led to a reduction in epithelial transparency. Additionally, investigations of human donor eyes have demonstrated minimal penetration of PVI in a time- and concentration-dependent manner into the epithelium, Bowman layer, and anterior and mid stroma. Therefore, after 5 min of immersion in 0.5% PVI, minimal iodine was observed in the epithelium. After 2 min, 2% PVI and 5% PVI could achieve only marginal penetration into the epithelium and Bowman layer and could not reach the stromal region [18]. Furthermore, the contradictory in vitro and ex vivo observations were also confirmed by investigations of PHMB performed by Valluri et al. [22]. Although PHMB exhibited potent activity against herpes simplex virus in vitro, this substance did not demonstrate the same virucidal effect at the same and commonly used concentrations in an in vivo rabbit model.

In summary, this study confirmed the cytotoxic effects of the antiseptic substances PVI and PHMB on cultured epithelial and conjunctival cells, previously observed using 2D cell culture models. However, in tissue-specific epithelial models as well as in porcine cornea models, these cytotoxic effects could not be confirmed. Therefore, the responses to the exposed substances in more complex tissue cultures are completely different from those in 2D cell culture systems. According to our observations, we concluded that 2D cell culture alone does not seem to be a suitable test model for the investigation of the potential pathological impacts of substances applied topically to the corneal epithelium. Three-dimensional constructs of the tissue-specific corneal epithelium as well as ex vivo cultured pig corneas are more suitable for reflecting the complexity of the corneal epithelium, including intercellular barriers such as tight junctions for selective movement of ions, macromolecules, pathogens, and other solutes across the epithelium. Using these models, we did not find evidence that the use of 1% PVI and 5% PVI for up to 2 min or 0.04% PHMB for up to 30 min correlates with possible wound-healing deficits after successful surgery.

## Data Availability

No new data were created.

## References

[1] Lindquist T.D., Maxwell A.J., Miller T.D., Win E.T., Novicki T., Fritsche T.R., Iliakis B., Montoya M. (2011). Preparation of corneal donor eyes comparing 1% versus 5% povidone-iodine. Cornea.

[2] Ristau T., Kirchhof B., Fauser S. (2014). Antisepsis with polyhexanide is effective against endophthalmitis after intravitreal injections. Acta Ophthalmol..

[3] Hansmann F., Kramer A., Ohgke H., Strobel H., Müller M., Geerling G. (2004). Polyhexamethylbiguanid (PHMB) as preoperative antiseptic for cataract surgery. Ophthalmologe.

[4] Silas M.R., Schroeder R.M., Thomson R.B., Myers W.G. (2017). Optimizing the antisepsis protocol: Effectiveness of 3 povidone-iodine 1.0% applications versus a single application of povidone-iodine 5.0. J. Cataract Refract. Surg..

[5] Jiang J., Wu M., Shen T. (2009). The toxic effect of different concentrations of povidone iodine on the rabbit’s cornea. Cutan. Ocul. Toxicol..

[6] Grzybowski A., Kanclerz P., Myers W.G. (2018). The use of povidone-iodine in ophthalmology. Curr. Opin. Ophthalmol..

[7] Shimada H., Nakashizuka H., Grzybowski A. (2017). Prevention and treatment of postoperative endophthalmitis using povidone-iodine. Curr. Pharm. Des..

[8] Wiegand C., Eberlein T., Andriessen A. (2017). Antibacterial activity of polihexanide formulations in a co-culture of HaCaT keratinocytes and Staphylococcus aureus and at different pH levels. Wound Repair Regen..

[9] Kim S., Ahn Y., Lee Y., Kim H. (2020). Toxicity of povidone-iodine to the ocular surface of rabbits. BMC Ophthalmol..

[10] Shibata Y., Tanaka Y., Tomita T., Taogoshi T., Kimura Y., Chikama T., Kihira K. (2014). Evaluation of corneal damage caused by iodine preparations using human corneal epithelial cells. Jpn. J. Ophthalmol..

[11] Papa V., van der Meulen I., Rottey S., Sallet G., Overweel J., Asero N., Minassian D.C., Dart J.K.G. (2022). Safety and tolerability of topical polyhexamethylene biguanide: A randomised clinical trial in healthy adult volunteers. Br. J. Ophthalmol..

[12] Castro N., Gillespie S.R., Bernstein A.M. (2019). Ex vivo corneal organ culture model for wound healing studies. J. Vis. Exp..

[13] Robertson D.M., Li L., Fisher S., Pearce V.P., Shay J.W., Wright W.E., Cavanagh H.D., Jester J.V. (2005). Characterization of growth and differentiation in a telomerase-immortalized human corneal epithelial cell line. Investig. Ophthalmol. Vis. Sci..

[14] Koerner J.C., George M.J., Meyer D.R., Rosco M.G., Habib M.M. (2018). Povidone-iodine concentration and dosing in cataract surgery. Surv. Ophthalmol..

[15] Barry P., Cordoves L., Gardner S. (2013). ESCRS Guidelines for Prevention and Treatment of Endophthalmitis following Cataract Surgery: Data Dilemmas and Conclusions. www.escrs.org/endophthalmitis/guidelines/ENGLISH.pdf.

[16] Shimada H., Nakashizuka H., Hattori T., Mori R., Mizutani Y., Yuzawa M. (2010). Effect of operative field irrigation on intraoperative bacterial contamination and postoperative endophthalmitis rates in 25-gauge vitrectomy. Retina.

[17] Yanai R., Yamada N., Ueda K., Tajiri M., Matsumoto T., Kido K., Nakamura S., Saito F., Nishida T. (2006). Evaluation of povidone-iodine as a disinfectant solution for contact lenses: Antimicrobial activity and cytotoxicity for corneal epithelial cells. Cont. Lens Anterior Eye.

[18] Pels E., Vrensen G.F. (1999). Microbial decontamination of human donor eyes with povidone-iodine: Penetration, toxicity, and effectiveness. Br. J. Ophthalmol..

[19] Lepelletier D., Maillard J.Y., Pozzetto B., Simon A. (2020). Povidone iodine: Properties, mechanisms of action, and role in infection control and staphylococcus aureus decolonization. Antimicrob. Agents Chemother..

[20] McDonnell G., Russell A.D. (1999). Antiseptics and disinfectants: Activity, action, and resistance. Clin. Microbiol. Rev..

[21] Sowlati-Hashjin S., Carbone P., Karttunen M. (2020). Insights into the polyhexamethylene biguanide (PHMB) mechanism of action on bacterial membrane and DNA: A molecular dynamics study. J. Phys. Chem. B.

[22] Valluri S., Fleming T.P., Laycock K.A., Tarle I.S., Goldberg M.A., Garcia-Ferrer F.J., Essary L.R., Pepose J.S. (1997). In vitro and in vivo effects of polyhexamethylene biguanide against herpes simplex virus infection. Cornea.

